# Novel Research in Low-Dimensional Systems

**DOI:** 10.3390/nano13020364

**Published:** 2023-01-16

**Authors:** Orion Ciftja

**Affiliations:** Department of Physics, Prairie View A&M University, Prairie View, TX 77446, USA; ogciftja@pvamu.edu

Low-dimensional systems exhibit unique properties that have attracted considerable attention during the last few decades. Notably, the fabrication of low-dimensional systems and devices with lengths that measure in the nanometer range has opened up investigations in a “new” type of science—nanoscience. A bulk three-dimensional system’s properties are typically insensitive to the size (as long as the size is macroscopic). However, all these considerations change when the size of such systems is reduced to the nanometer range. A known fact is that, unlike their bulk counterparts, many low-dimensional systems tend to exhibit novel and unique phenomena of great interest to many scientific disciplines. Furthermore, in the case of nanostructures, many of them manifest size-dependent properties, as well as behaviors that are strongly dictated by the rules of quantum mechanics. Therefore, understanding their properties is both highly interesting and rewarding because of the various possible technological applications. A great deal of progress has been achieved in the field of material science, including the fabrication of novel materials with length scales in the nanometer range [1,2,3,4,5,6]. Systems such as carbon nanotubes, nanowires, quantum dots, thin films, etc., manifest amazing properties and are already featured in several emerging technologies and advanced applications. The application of new and extraordinary experimental tools in the field has created an urgent need for an improved understanding of the new physical phenomena that occur in such low-dimensional systems. This has drawn the interest of many experimental and theoretical groups around the world [7,8,9,10,11,12]. The aim of this Special Issue is to provide an overview of the current research in low-dimensional systems by attracting contributions from specialists in the field. This way, we try to provide important insights on the large variety of scientifically fascinating and technologically important phenomena that are being investigated. The covered topics include original research articles on the fundamental and applied aspects of physics in various low-dimensional systems, such as quantum dots, graphene nanosystems, ultrathin films, superconducting nanofilms, novel nanoscale devices, etc. The present Special Issue includes research papers from both theoretical and experimental groups, with many phenomena studied from a multi-disciplinary perspective. There are 10 research papers in this Special Issue, which explore important developments in the field of low-dimensional systems.

The first paper by Metzke et al. [13] illustrates the use of atomic force microscopy (AFM)-based scanning thermal microscopy techniques to characterize the thermal properties of nanoscale systems. Specifically speaking, this work focuses on theoretical studies of ultrathin films with anisotropic thermal properties, such as hexagonal boron nitride (h-BN), and compares the results with a bulk silicon (Si) sample. The second paper by Kapcia [14] investigates the charge order on triangular lattices for fermionic particles that are described by an extended Hubbard model. A triangular lattice is formed by a single layer of graphene or graphite surfaces, as well as the (111) surface of face-centered cubic crystals.The author uses an extension of the lattice gas model for S=1/2 fermionic particles on a two-dimensional triangular (hexagonal) lattice to analyze the system within the mean field approximation. The model’s qualitative differences considering hypercubic lattices are also discussed.The third paper by Ciftja [15] represents a theoretical study of a nanocapacitor’s electric properties. Such properties can be very different from the expected bulk properties due to the finite-size effects for small length scales. Additionally, a theoretical model for a circular parallel-plate nanocapacitor is considered, and analytic expressions for the nanocapacitor’s stored electrostatic energy and capacitance are derived. The obtained results can be easily used to incorporate the effects of a dielectric thin film in case the space between the circular plates of the nanocapacitor is filled with such a film. The fourth paper by Wu et al. [16] considers a graphene nanoribbon gap waveguide as a candidate system for guiding dispersionless gap surface-plasmon polaritons with deep-subwavelength confinement and low loss. An analytical model is developed to analyze the system, in which a reflection phase shift is employed to successfully deal with the influence caused by the boundaries of the graphene nanoribbon. The proposed setup may be of great interest in studying dispersionless and low-loss nanophotonic devices and may have various potential technological applications. The fifth paper by Du et al. [17] focuses on the properties of graphene-based nanocomposite films. Nanocomposite films of this nature are in high demand due to their superior photoelectric and thermal properties; however, their stability and mechanical properties pose challenges. Motivated by these facts, this work illustrates a facile approach that can be used to prepare various nanocomposite films through the non-covalent self-assembly of graphene oxide and biocompatible proteins. Various characterization techniques were employed to characterize the properties of such nanocomposites and to track the interactions between graphene oxide and proteins. It is suggested that this strategy should be facile and effective for fabricating well-designed bio-nanocomposites for universal functional applications. The sixth paper by Wang et al. [18] reports the findings of a study on the influence of ink properties on the morphology of long-wave infrared HgSe quantum-dot films. The main focus of the analysis are the various factors affecting the morphology of the films, including ink surface tension, particle size, and solute volume fraction. This work is important for the morphology control of the filter film arrays, which are core components to many optoelectronic devices and for detecting targets by spectroscopic methods. The various system properties were analyzed in terms of different changing variables. The seventh paper by Alotabi et al. [19] studies the effect of TiO_2_ film thickness on the stability of Au_9_ clusters with a CrO*_x_* layer. The high-purity TiO_2_ films are fabricated via radio-frequency magnetron sputtering techniques, which allow the reliable control of film thickness and uniform morphology. The change in surface roughness upon heating two TiO_2_ films with different thicknesses was investigated. Chemically-synthesized phosphine-protected Au_9_ clusters covered by a photo-deposited CrO*_x_* layer were used as a probe. It was found that the high mobility of the thick TiO_2_ film after heating leads to a significant agglomeration of the Au_9_ clusters, even when protected by the CrO*_x_* layer. The eighth paper by Abramkin and Atuchin [20] is a theoretical analysis of hole states energy spectra in novel InGaSb/AlP self-assembled III-V quantum dots. These materials may have possible applications in non-volatile memories. Material intermixing and the formation of strained structures were also taken into account. The authors found that adjusting the values of various parameters allows one to find an optimal device configuration for possible non-volatile memory applications. The search for novel self-assembled quantum dots with hole-localization energy that allow a long charge storage is very important to the field of non-volatile memory applications. The ninth paper by McNaughton et al. [21] studies the causes and consequences of ordering and the dynamic phases of confined vortex rows in superconducting nanostripes. Superconducting nanostripes are a fundamental component in superconducting electronics, and they are crucial components for various applications in the field of quantum technology. Therefore, understanding the behavior of vortices under nanoscale confinement in superconducting circuits is important for the development of superconducting electronics and quantum technologies. Numerical simulations based on the Ginzburg–Landau theory for non-homogeneous superconductivity in the presence of magnetic fields are also carried out. The findings lead to the understanding of how lateral confinement organises vortices in a long superconducting nanostripe. A phase diagram of vortex configurations as a function of the stripe width and magnetic field is also presented. The tenth paper by Sharma et al. [22] sheds light on complex phase-fluctuation effects correlated with granularity in superconducting NbN nanofilms. Superconducting nanofilms are tunable systems that can result in the Berezinskii–Kosterlitz–Thouless superconducting transition when the system approaches the two-dimensional regime. Reducing the dimensionality further to quasi one-dimensional superconducting nanostructures with disorder can generate quantum and thermal phase slips of the order parameter. Experimental studies of these phenomena are difficult. As a result, the characterization of superconducting NbN nanofilms under different conditions carried out in this study can be very useful for future work.
